# Assessing the prevalence of extensive macular atrophy with pseudodrusen-like appearance in patients with rheumatic fever-associated valvular heart disease: a cross-sectional study

**DOI:** 10.1186/s40942-026-00805-6

**Published:** 2026-02-02

**Authors:** Antonio S. F. Cassavia Junior, Victor Bellanda, Leticia O. Audi, Gabriel Castilho S. Barbosa, Renata A. M. Caravelas, Gustavo J. Volpe, Henrique T. Moreira, Andre Schmidt, Rodrigo Jorge

**Affiliations:** 1 Ophthalmology Division, Ribeirão Preto Medical School, University of São Paulo, Ribeirão Preto, SP Brazil; 2 Cole Eye Institute, Cleveland Clinic, Cleveland, OH USA; 3 Department of Ophthalmology, University of São Paulo, São Paulo, SP Brazil; 4 Cardiology Division, Internal Medicine Department, Ribeirão Preto Medical School, University of São Paulo, Ribeirão Preto, SP Brazil

**Keywords:** Age-related macular degeneration, Subretinal drusenoid deposits, Rheumatic fever, Risk factors, Epidemiology

## Abstract

**Background:**

Extensive macular atrophy with pseudodrusen-like appearance (EMAP) is a rare retinal disorder characterized by bilateral macular atrophy and subretinal drusenoid deposits (SDDs), typically occurring earlier and progressing faster than age-related macular degeneration. A possible association between EMAP and rheumatic fever (RF) has been proposed, but its prevalence in this population remains unclear.

**Methods:**

In this cross-sectional study, 118 patients (236 eyes) with valvular disease secondary to RF were prospectively screened at a tertiary cardiology clinic. EMAP was defined by vertically predominant macular atrophy with SDDs and exclusion of alternative diagnoses. Multimodal imaging included spectral-domain optical coherence tomography and fundus autofluorescence. Demographic, clinical, and cardiologic parameters were descriptively analyzed between patients with and without EMAP, and compared between those with and without SDDs using appropriate statistical tests.

**Results:**

Two patients (1.69%; 95% confidence interval [CI], 0.47–5.97%) met diagnostic criteria for EMAP. Both were women aged 62 and 73 years, with RF onset during childhood and long disease duration (54 and 62 years). SDDs were identified in 12 patients (10.17%; 95% CI, 5.91–16.94%), including both EMAP cases. Patients with SDDs were significantly older (*p* = 0.007), had longer RF duration (*p* = 0.032), and received a greater cumulative benzathine penicillin prophylaxis burden (*p* = 0.029).

**Conclusions:**

EMAP is uncommon among patients with RF-associated valvular disease, suggesting that additional genetic or environmental factors may be necessary for disease manifestation. The prevalence of SDDs highlights potential subclinical retinal involvement associated with chronic systemic inflammation in this patient population.

**Supplementary Information:**

The online version contains supplementary material available at 10.1186/s40942-026-00805-6.

## Introduction

Extensive macular atrophy with pseudodrusen-like appearance (EMAP) is a rare clinical entity first described by Hamel et al. in 2009 [1]. It is characterized by extensive bilateral macular atrophy with a predominant vertical axis, mid-peripheral pseudodrusen-like deposits, and paving stone degeneration in the far periphery [2]. Unlike dry age-related macular degeneration (AMD), EMAP typically manifests before the age of 55, progresses rapidly, and leads to significant central visual deterioration within a few years, although diagnoses at older ages do occur and have contributed to past misclassification as AMD [1, 3, 4]. Multimodal imaging reveals hallmark features such as faint, multilobular hypo-autofluorescence on short-wavelength autofluorescence and a diffuse separation between Bruch’s membrane (BM) and the retinal pigment epithelium (RPE) on optical coherence tomography (OCT) [5]. These distinct characteristics underscore the need for accurate differentiation from AMD and other macular atrophies.

Recent studies have highlighted a potential link between EMAP and rheumatic fever (RF), an inflammatory autoimmune disease caused by group A β-hemolytic streptococcal infections [6–8]. In a cohort of Brazilian patients, over 90% of individuals diagnosed with EMAP reported a history of RF, with 94.8% undergoing long-term benzathine penicillin (BP) prophylaxis [7]. These findings suggest that chronic immune responses triggered by RF and potentially exacerbated by prolonged BP use may play a role in the pathogenesis of EMAP.

The importance of investigating this association extends beyond regional contexts, as EMAP remains an underdiagnosed condition, particularly in older individuals, who are often misdiagnosed with AMD [9–12]. Identifying the phenotypic and structural markers of EMAP could enhance diagnostic accuracy and deepen our understanding of its underlying mechanisms. Furthermore, exploring the intersection of RF, BP use, and macular atrophy offers valuable insights into the immunopathological processes that may contribute to retinal degeneration.

Given the severity of early visual loss and the likely inflammatory and immune-mediated mechanisms underlying this aggressive form of macular degeneration, this study aimed to actively screen patients with a history of RF who developed cardiac disease, identifying signs of EMAP and characterizing the affected patient population.

## Methods

This cross-sectional study assessed the presence of findings consistent with EMAP in patients with a history of RF and secondary valvulopathy. Patients diagnosed with valvular disease secondary to RF were recruited from the cardiology department at *Hospital das Clínicas de Ribeirão Preto* (HCRP), a tertiary hospital affiliated with the University of São Paulo, Brazil. All participants provided written informed consent before enrolment. The study protocol was approved by the HCRP Institutional Review Board (approval number 7171762; date of approval: 10/2/2024), and all procedures adhered to the ethical principles of the Declaration of Helsinki.

A total of 264 patients from the cardiology valvular disease outpatient clinic were invited for a comprehensive ophthalmic evaluation at the Retina and Vitreous outpatient clinic on a separate date from their original cardiology appointment, from March to December of 2024. Inclusion criteria comprised individuals aged ≥ 18 years with a confirmed diagnosis of valvular disease secondary to RF, adequate pupillary dilation, clear ocular media, and sufficient cooperation for ophthalmic imaging. Patients were excluded if they had significant media opacity hindering posterior segment examination, inability to undergo pupillary dilation, mobility limitations preventing imaging, or severe intellectual disability precluding informed consent. Additional exclusion criteria included the presence of other retinal, vitreous, or choroidal diseases affecting visual function, systemic conditions interfering with imaging or follow-up, or any medical or psychological impairment preventing study participation.

Of the 264 invited patients, 118 (44.7%) consented to participate and attended the ophthalmic visit. The remaining 146 individuals either declined participation or did not attend the scheduled examination. All 118 participants underwent a comprehensive ophthalmic evaluation, which included best-corrected visual acuity (BCVA) assessment using a logMAR ETDRS chart, anterior and posterior segment biomicroscopy with a slit lamp and a 78-diopter Volk lens (Volk, Germany), and indirect binocular ophthalmoscopy with a 20-diopter Volk lens. Multimodal retinal imaging was performed, including fundus photography, spectral-domain OCT and fundus autofluorescence (FAF) using the Spectralis^®^ HRA + OCT system (Heidelberg Engineering, Germany). All imaging procedures were conducted under standardized conditions to ensure reproducibility and minimize patient discomfort. Full-field and multifocal electroretinograms (ERG) were performed according to ISCEV (International Society for Clinical Electrophysiology of Vision) standards.

Patients were assessed for the presence of drusen, subretinal drusenoid deposits (SDDs; pseudodrusen), macular atrophy, horizontal and vertical axis of atrophy, presence of a neovascular membrane, and peripheral pavingstone-like degeneration. No participants were excluded following the ophthalmologic examination, as none met the exclusion criteria. The primary outcome was the identification of cases consistent with EMAP, defined by an adaptation of the criteria proposed by Antropoli et al. [13]:

1. Presence of SDDs and pseudodrusen-like deposits involving the posterior pole and midperiphery on fundus examination and multimodal *en face* imaging;
2. At least one of the following: (a) no evident macular atrophy on SW-AF but diffuse macular RPE-BM separation on OCT; (b) Moderately hypoautofluorescent (“grayish”) macular atrophy on autofluorescence surrounded by RPE-BM separation on OCT; or (c) Moderately hypoautofluorescent (“grayish”), multilobular macular atrophy with vertical, “leaf-shaped”, or confluent configuration along with “temporal sparing”;
3. Absence of any other diagnosis that could better explain these findings.

 Secondary outcomes included the presence of drusen and SDDs detected on OCT, near-infrared reflectance (NIR), and FAF.

A comprehensive chart review was conducted for all included patients to collect demographic and clinical data, including age, sex, history and duration of BP prophylaxis, age at RF diagnosis, disease duration, type of valvulopathy, and the most recent ejection fraction assessed by echocardiography. These details were further supplemented through patient interviews conducted at the time of the physical exam.

Statistical analyses included descriptive statistics to summarize the demographic and clinical characteristics of patients with and without EMAP, as well as those with and without SDDs. Categorical variables, such as sex and BP use, were expressed as frequencies and percentages, while continuous variables, including age, disease duration, and duration of penicillin use, were reported as means with standard deviations (SD) when normally distributed, and medians with interquartile ranges (IQR) when non-normally distributed. Group comparisons were conducted to explore potential associations between the prevalence of SDDs and clinical factors such as the duration of rheumatic heart disease and BP prophylaxis. The Shapiro-Wilk test was performed to assess for normality of data distribution in each variable. For normally distributed continuous variables, Student’s t-test was employed. Non-normally distributed continuous variables were analyzed using the Mann-Whitney U test (Wilcoxon rank-sum test), and categorical variables were compared using Fisher’s exact test. The 95% confidence intervals (CI) for the prevalence of EMAP and SDDs were calculated using the Wilson score interval. Statistical analyses were performed with Python 3.12. Statistical significance was set at *p* < 0.05 where applicable.

## Results

A total of 118 patients (236 eyes) were evaluated between April 2024 and December 2024. The mean patient age was 56.5 ± 12.1 years, with a predominance of female participants (99 women, 83.9%). All patients had a history of valvular disease secondary to RF, with a median age at RF diagnosis of 11.0 ± 7.0 years and an average disease duration of 42.4 ± 14.4 years. Among the cohort, 105 patients (89.0%) reported prior use of BP, with a median duration of 14.0 ± 20.0 years.

Two cases met the diagnostic criteria for EMAP, presenting with macular atrophy associated with SDDs in the posterior pole and mid-periphery, following a predominantly vertical distribution. This accounted for 1.69% of our sample, with an estimated 95% CI for the general prevalence among the RF population ranging from 0.47% to 5.97%. These cases are discussed in further detail below. Twelve patients (10.17%; 95% CI 5.91%-16.94%) exhibited SDDs in the posterior pole, including two cases diagnosed with EMAP, all confirmed by OCT and FAF imaging. Drusen were present in 23 patients (19.49%; 95% CI 13.35%-27.55%), of whom 10 patients (8.47%; 95% CI 4.67%-14.90%) had findings consistent with non-exudative AMD. Additionally, one patient exhibited features of a previously exudative form of AMD but presented with a disciform scar at the time of examination.

Case 1 was a 73-year-old woman who had acute RF at the age of 11 and used prophylactic BP every 15 days for two years after her diagnosis, and sporadically on her 20s. She was followed up at the cardiology clinic since 2013 due to aortic stenosis and mitral stenosis. Her co-morbidities included chronic atrial flutter, for which she used oral anticoagulation with warfarin, obesity, and stage II chronic kidney disease with right renal atrophy. She had mitral commissurotomy at age 33, valvuloplasty at age 43, mitral valve replacement and tricuspid valve replacement at age 48. At the time of the last visit, she was NYHA Class III. The patient reported low visual acuity noticed over ten years ago. She was followed up for a short period of time in an ophthalmology clinic around seven years ago and had a diagnosis of dry AMD with geographic atrophy in both eyes. She did not take AREDS vitamins or did any type of treatment and she did not follow up her condition with regular exams.

**Fig. 1:**
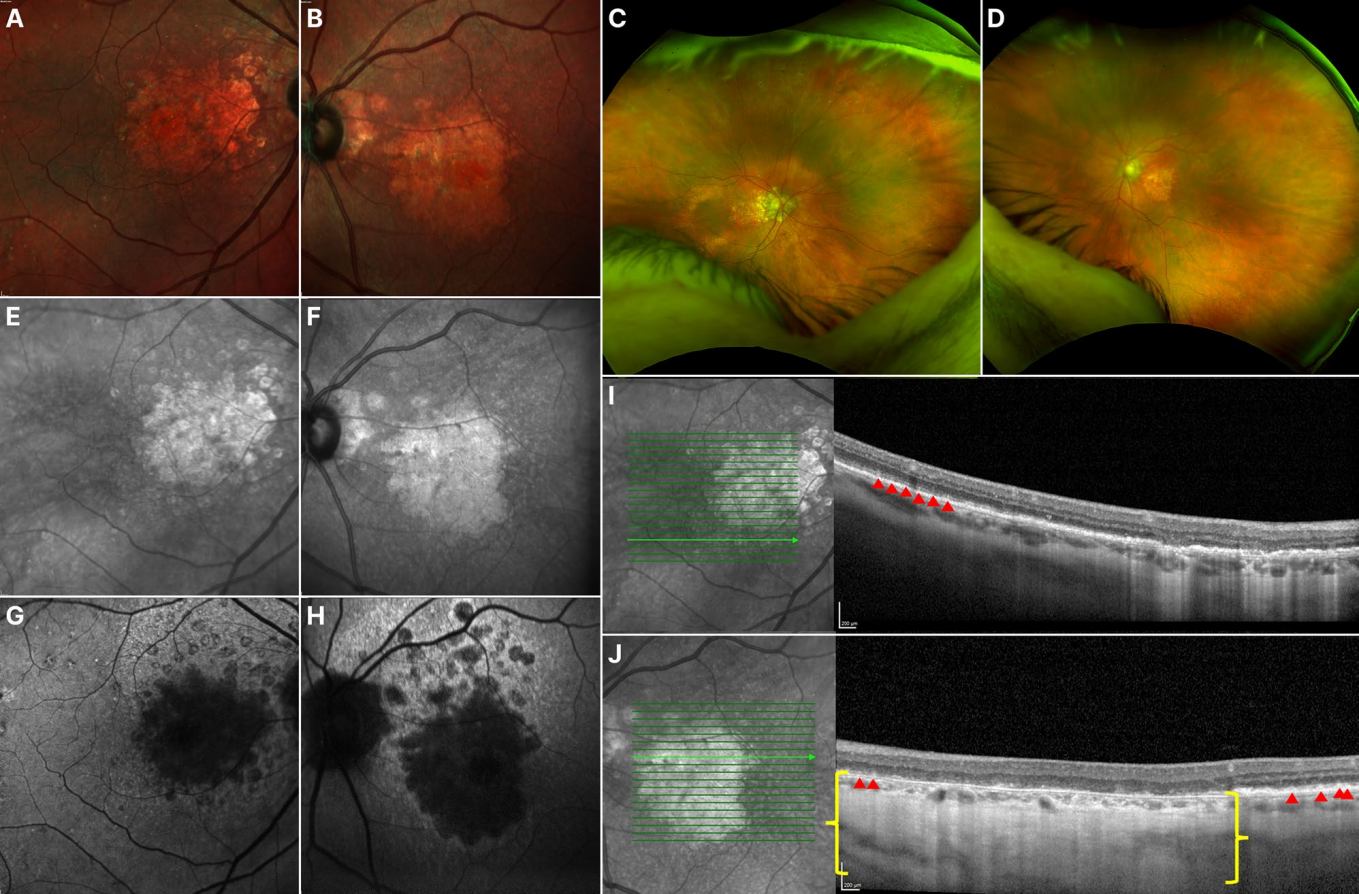
Multimodal imaging of Case 1. Fundus photographs of the OD (**A**) and OS (**B**) show central macular atrophy, with vertical axis greater than the horizontal in the OS, pigmentary changes, and diffuse pseudodrusen-like deposits in the posterior pole OU. Ultra-widefield fundus photographs of the OD (**C**) and OS (**D**) show subtle pavingstone-like degeneration in the periphery. Near-infrared reflectance imaging (**E-F**) confirms with higher contrast the findings of the fundus photographs. Fundus autofluorescence (**G-H**) shows a central hypo-autofluorescent lesion consistent with macular atrophy, surrounded by areas of focal atrophic lesions reaching the superior arcade. OCT b-scans of the OD (**I**) and OS (**J**) show sparse subretinal drusenoid deposits (red arrowheads), diffuse ellipsoid zone attenuation, complete retinal pigment epithelium and outer retinal atrophy (cRORA) (yellow brackets), and diffuse choroidal thinning

On ophthalmic assessment on our study, she presented with scotomas and central metamorphopsia on the Amsler grid in both eyes. BCVA was 20/160 OD and counting fingers at 3 feet OS. Biomicroscopy showed no relevant findings OU. Funds exam was significant for macular atrophy OU, with a vertical axis higher than the horizontal in the OS. The atrophy included the foveal region, with perifoveal paving-stone-like changes. OCT revealed sparse drusen and SDDs, predominantly in the macula, along with diffuse attenuation of the ellipsoid zone (EZ), complete RPE and outer retinal atrophy (cRORA), and diffuse choroidal thinning. FAF revealed signs of central RPE atrophy, represented by a hypo-autofluorescent area with scalloped, iso-autofluorescent borders, which suggests more advanced disease [14]. NIR imaging confirmed the pattern of foveal-involving RPE atrophy (Fig. 1).

Full-field ERG responses were within normal ranges for amplitude and latency. However, in the OS, which showed the greatest anatomical involvement on OCT, there was a reduction in b-wave amplitudes across all scotopic stimuli, a decreased peak-to-peak amplitude in response to the 30 Hz flicker stimulus, and a reduction in a-wave amplitudes under both scotopic and photopic conditions compared to the OD. The electrophysiological assessment was complemented by multifocal ERG, which revealed reduced macular amplitude in both eyes, with a particularly pronounced decrease around the macula in the OS (Supplementary File 1).

 Case 2 was a 62-year-old woman diagnosed with double mitral valve disease, with severe mitral regurgitation and moderate mitral stenosis. She also had mild to moderate aortic regurgitation and secondary tricuspid regurgitation. She had been on anticoagulation therapy with warfarin for the past two months due to atrial fibrillation. The patient had been followed in the cardiology outpatient clinic since 2012. She was diagnosed with acute RF at the age of 8 and received prophylactic BP every 21 days from ages 8 to 40. Despite these conditions, she did not report any ophthalmologic complaints.

**Fig. 2:**
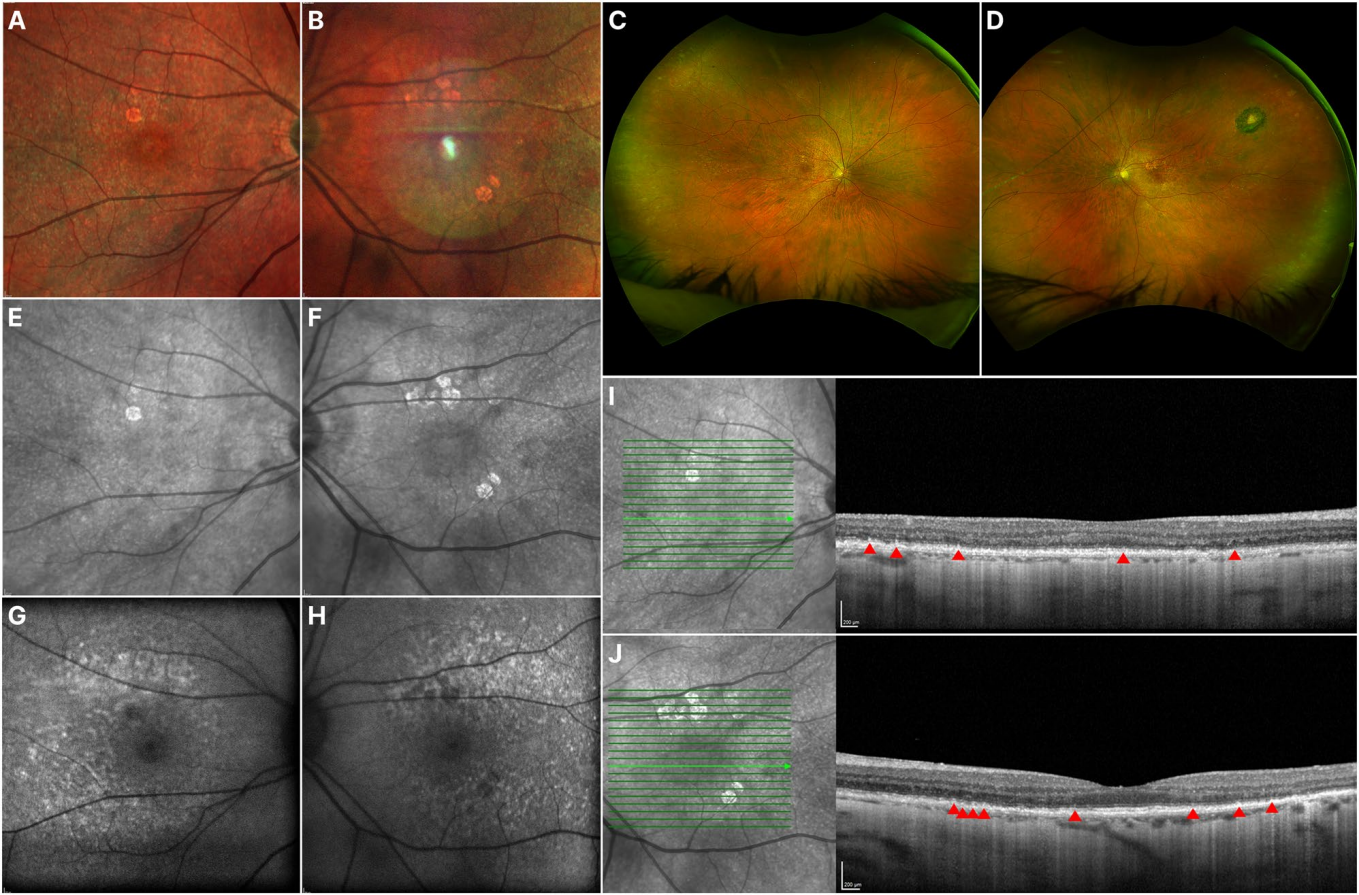
Multimodal imaging of Case 2. Fundus photographs of the OD (**A**) and OS (**B**) show focal areas of macular atrophy and pigmentary changes, in addition to diffuse pseudodrusen-like deposits in the posterior pole. The fundus photograph of the OS (**B**) also shows a round light artifact. Ultra-widefield fundus photographs of the OD (**C**) and OS (**D**) show peripheral pavingstone-like degeneration, and an atrophic chorioretinal scar superotemporal to the macula in the OS. Near-infrared reflectance imaging (**E-F**) highlights the focal areas of atrophy. Fundus autofluorescence (**G-H**) shows a confluent pattern of hypo-autofluorescent punctate lesions, sparing the fovea. OCT b-scans of the OD (**I**) and OS (**J**) show sparse subretinal drusenoid deposits (SDDs) (red arrowheads), incomplete retinal pigment epithelium and outer retinal atrophy (iRORA) represented by the barcode sign, diffuse prominent choroidal thinning, and areas of retinal pigment epithelium-Bruch’s membrane separation surrounding the macular atrophy

On exam, BCVA was 20/40 OU. Biomicroscopy showed no relevant findings. Fundus exam was significant for focal areas of atrophy in the macula OU, more predominant in the OS, along with pigmentary changes. OCT revealed sparse drusen and SDDs, with preserved EZ in the fovea, along with incomplete RPE and outer retinal atrophy (iRORA) OU, and focal areas of cRORA in the OS. FAF revealed coalescent hyperautofluorescent areas with foveal sparing, characteristic of early-stage disease, and focal hypo-autofluorescent areas corresponding to RPE atrophy. NIR imaging depicted the same focal areas of RPE atrophy, along diffuse punctate hyper-reflective lesions (Fig. 2).

ERG of the OS, which was the more severely affected, showed reduced b-wave amplitude in response to scotopic stimuli and reduced a-wave amplitude under photopic conditions. Additionally, multifocal ERG revealed a significant reduction in central amplitude in both eyes. These findings are consistent with OCT and autofluorescence, indicating involvement of the entire foveal region (Supplementary File 2).

**Fig. 3:**
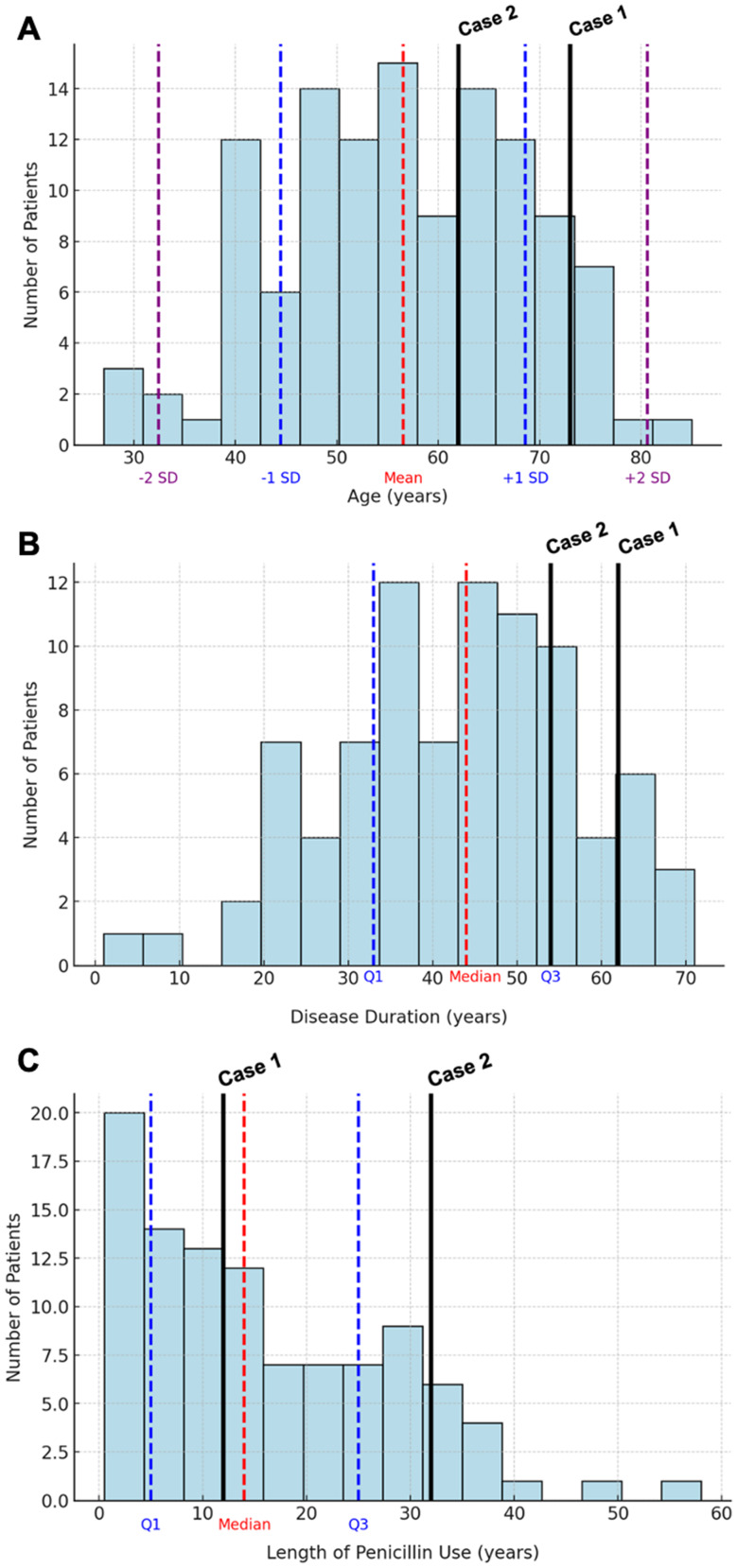
Histogram of the number of patients by age (**A**), disease duration (**B**), and length of penicillin use (**C**), highlighting the two cases of EMAP found in our sample. SD – Standard Deviation; Q – Quartile

Table 1 compares the clinical characteristics of the two patients diagnosed with EMAP to the rest of the cohort. The small number of EMAP cases in our cohort precluded any statistically significant comparisons. However, these patients were notably older (73 and 62 years vs. 56.3 ± 12.1 in the rest of the cohort) and had a longer disease duration (62 and 54 years vs. 42.4 ± 14.4). The duration of BP use was comparable between groups (12 and 32 years vs. 14.0 ± 20.0) (Fig. 3).

**Table 1:** Comparison between patients with EMAP and the remaining cohort

	NO EMAP (*n* = 116)	EMAP Case 1	EMAP Case 2
**Age**,** years** (mean ± SD)	56.3 ± 12.1	73.0	64.0
**Age at diagnosis**, years* (median [IQR])	11.0 [7.0]	11.0	8.0
**Female sex**, n (%)	97 (83.6%)	Yes	Yes
**Duration of disease**, years* (mean ± SD)	42.1 ± 14.4	62.0	54
**BP prophylaxis**, n (%)	103 (88.8%)	Yes	Yes
**Duration of BP prophylaxis**, years** (median [IQR])	14.0 [20.0]	12.0	32.0
**Ejection fraction**, % (median [IQR])	56.5 [10.0]	63.0	64.0
**BCVA OD**, logMAR (median [IQR])	0.80 [0.40]	0.90	0.30
**BCVA OS**, logMAR (median [IQR])	0.80 [0.40]	2.30	0.30

Abbreviations: EMAP – Extensive Macular Atrophy with Pseudodrusen-like Appearance; SD – Standard Deviation; IQR – Interquartile Range; BP – Benzathine Penicillin; BCVA – Best Corrected Visual Acuity; OD – right eye; OS – left eye

*Data available for 87 patients (2 with EMAP, 85 without EMAP)

**Data available for 103 patients (2 with EMAP, 101 without EMAP)

Statistical analysis examining the association between SDDs and demographic/clinical factors (Table 2) revealed a significant correlation with age (*p* = 0.007), disease duration since RF diagnosis (*p* = 0.032), and cumulative burden of BP use (*p* = 0.029). However, no significant associations were found between SDD presence and valvular disease type (Supplementary File 3) or ejection fraction.

**Table 2:** Comparison between patients with and without SDDs

	SDDs (*n* = 12)	NO SDDs (*n* = 106)	*p*-value	Test used
**Age**,** years** (mean ± SD)	65.4 ± 7.4	55.5 ± 12.2	**0.007**	t-test
**Age at diagnosis**, years* (median [IQR])	9.5 [3.0]	11.0 [7.0]	0.589	Mann-Whitney U
**Female sex**, n (%)	11 (91.7%)	88 (83.0%)	0.688	Fischer’s
**Duration of disease**, years* (mean ± SD)	51.6 ± 9.9	41.2 ± 14.5	**0.032**	t-test
**Patients with BP prophylaxis**, n (%)	12 (100%)	93 (87.7%)	0.357	Fischer’s
**Duration of BP prophylaxis**, years** (median [IQR])	25.0 [20.75]	13.0 [17.5]	**0.029**	Mann-Whitney U
**Ejection fraction**, % (median [IQR])	58.5 [12.75]	56.0 [9.0]	0.799	Mann-Whitney U
**BCVA OD**, logMAR (median [IQR])	0.66 [0.46]	0.80 [0.40]	0.019	Mann-Whitney U
**BCVA OS**, logMAR (median [IQR])	0.73 [0.33]	0.80 [0.40]	0.234	Mann-Whitney U

Abbreviations: SDDs – Subretinal Drusenoid Deposits (pseudodrusen); SD – Standard Deviation; IQR – Interquartile Range; BP – Benzathine Penicillin; BCVA – Best Corrected Visual Acuity; OD – right eye; OS – left eye

*Data available for 87 patients (2 with SDD, 85 without SDD)

**Data available for 103 patients (12 with SDD, 91 without SDD)

## Discussion

This is the first study to systematically assess the prevalence of EMAP in patients with a history of rheumatic valvular disease. Among 118 patients evaluated, only two cases met the diagnostic criteria for EMAP, reinforcing its rarity even in a potentially predisposed population. While our results indicate that EMAP is not a common finding in patients with rheumatic valvular disease, they do not refute this previously proposed association but rather contextualize it within a broader epidemiological framework.

In a study that analyzed a cohort of EMAP patients in Brazil, over 90% of subjects reported a history of RF and long-term BP prophylaxis (average duration of 11.8 years), suggesting that chronic immune dysregulation and prolonged exposure to BP may play a role in disease pathogenesis [7]. In our cohort, the length of BP use was longer among patients with SDDs but remained comparable between those with and without EMAP. The potential contribution of prolonged BP use remains hypothetical, and no causal mechanism has been established. Notably, Moreira-Neto et al. found that longer BP treatment duration was paradoxically associated with smaller atrophic areas and a reduced vertical axis of atrophy in EMAP-like eyes, suggesting that prolonged BP use may not exacerbate retinal degeneration and could even be associated with less extensive atrophy in some patients [7]. Given that RF triggers a sustained inflammatory response leading to both cardiac and systemic sequelae, it has been hypothesized that chronic immune activation, molecular mimicry, and potential antibiotic-induced alterations in the intestinal microbiome may contribute to retinal degeneration in susceptible individuals [6, 7, 15]. In accordance with this hypothesis, immunological cross-reactivity of two monoclonal antibodies against the streptococcal M protein with the outer and inner segments of the retina has been reported [16]. These concepts, however, remain exploratory, and further mechanistic and longitudinal studies are needed to clarify them.

In addition to structural degeneration, functional evidence supports diffuse retinal involvement in EMAP. A recent electrophysiological study by Watanabe et al. demonstrated marked reduction of the photopic negative response, indicating early and severe retinal ganglion cell dysfunction even in patients with preserved visual acuity and relatively intact cone-mediated responses [8]. 

A study comparing patients with EMAP to healthy controls in France found a correlation between EMAP and an abnormal erythrocyte segmentation rate, reduced CH50 and increased plasma C3 levels [3]. These findings suggest that a delayed immunogenic response may be occurring in these patients years after the initial streptococcal infection. The same study, however, found no correlation between EMAP and cardiac diseases including heart failure, myocardial infarction, and cardiac rhythm disorders. This should be interpreted in the context of the much lower prevalence of rheumatic heart disease in France compared with Brazil, which may have resulted in an insufficient number of patients with RF-related cardiac disease to influence the results [17]. 

The presence of SDDs, a hallmark of EMAP, suggests a possible choroidal, RPE, or photoreceptor dysfunction as part of this disease spectrum. SDD prevalence increases with age and is notably higher in individuals with AMD. Zarubina et al. reported an overall prevalence of 32% in individuals aged ≥ 60 years, with rates of 23% in those without AMD and 52% in those with AMD [18]. The Montrachet Study [19] found an SDD prevalence of 18.1% in an elderly population (mean age: 82.3 years), while the Rotterdam Study [20] reported a lower prevalence of 4.9% in individuals aged ≥ 65 years using near-infrared imaging. In our cohort, nine out of 49 patients aged ≥ 60 years (18.37%) exhibited SDDs, a prevalence comparable to that observed in the general elderly population. However, SDDs have also been associated with cardiovascular disease and chronic systemic inflammation [21, 22]. In our study population, chronic systemic inflammation and cardiovascular disease may have contributed to their pathogenesis, in addition to aging itself.

Macular atrophy in degenerative retinal diseases typically emerges after age 65, progressing from perifoveal involvement to foveal atrophy [13]. In our cohort, the two EMAP cases followed this pattern: Case 2 (62 years old) had perifoveal atrophy with foveal sparing, while Case 1 (73 years old) exhibited advanced foveal involvement. Notably, despite Case 1 having a shorter BP exposure, both patients were older and had a longer disease duration compared to the average rest of the cohort (73 and 62 years vs. 56.3 ± 12.1; 62 and 54 years of disease vs. 42.4 ± 14.4). Their cumulative BP exposure was comparable to other participants (12 and 32 years vs. 14.0 ± 20.0), suggesting that age and disease chronicity may be stronger determinants of macular atrophy severity than BP use alone. This supports the hypothesis that, although ageing is not a causative factor in EMAP, older age and longer disease duration likely influence the degree of atrophy at presentation, with older individuals being more prone to widespread atrophic changes.

A recent ultrawidefield imaging study demonstrated that peripheral involvement in EMAP is highly variable, ranging from subtle RPE alterations to extensive paving stone–like or pigmented chorioretinal atrophy, with substantial heterogeneity in both severity and progression among patients [23]. Both EMAP cases in our cohort showed little to no peripheral atrophy, aligning with the “predominantly central pattern” described by Battaglia Parodi et al. This variability remains incompletely understood, and it is unclear whether these differences represent distinct phenotypes or different stages of the same disease. Our findings therefore fall within the expected phenotypic spectrum and highlight that not all EMAP cases exhibit marked extramacular involvement at presentation [23]. 

A critical distinction between our study and previous reports is our methodological approach. While prior studies analyzed patients with established EMAP and retrospectively identified RF as a common comorbidity, our study prospectively evaluated a subset of RF patients with the explicit objective of determining EMAP prevalence. This fundamental difference in study design explains the disparity in findings. The relatively low prevalence observed in our cohort suggests that not every RF patient is at risk for EMAP, reinforcing the notion that additional genetic, environmental, or immunologic factors may be necessary for disease manifestation.

Although EMAP was rare in our actively screened cohort (1.69%), the presence of SDDs in 10.17% of patients, rising to 18.37% among those aged ≥ 60, suggests that a subset of RF patients may exhibit early or subclinical retinal alterations. Given the significant associations between SDDs and age, disease duration, and cumulative BP exposure, targeted ophthalmologic evaluation using multimodal imaging may be beneficial in selected high-risk individuals, particularly older patients or those with visual symptoms. Future studies should include age-matched control groups, longitudinal follow-up to track SDD progression, and integration of immunological profiling to clarify whether SDDs in RF patients represent a distinct phenotype or a potential early stage in the EMAP spectrum.

Our study has several limitations. First, despite its cross-sectional design, the sample size remains relatively small, limiting statistical power to detect rare associations. Second, our cohort was derived from a tertiary cardiology center and only 45% of invited patients agreed to participate, which may not fully represent the broader RF population and introduces a potential selection bias. Third, while we employed comprehensive multimodal imaging to assess for EMAP features, subclinical cases may have been overlooked, especially in early-stage disease. Fourth, the diagnostic criteria proposed by Antropoli et al. include genetic testing to rule out inherited retinal dystrophies, a resource that was not available within the Brazilian public health system, where this study was conducted. Lastly, our study did not include a control group of age-matched individuals without rheumatic valvular disease, which would have strengthened the comparative analysis.

Despite these limitations, our findings provide valuable insight into the epidemiology of EMAP and its relationship with RF. By demonstrating a low prevalence of EMAP within this specific high-risk subset, we contribute essential data to refine the understanding of this rare entity. Importantly, our results highlight the need for future investigations to delineate the precise pathogenic mechanisms linking RF, BP use, and EMAP, explore potential genetic predispositions, and identify biomarkers that could aid in early detection. This study serves as a foundation for further research, emphasizing that while most EMAP patients in Brazil have a history of RF, the inverse is not necessarily true.

## Supplementary Information

Below is the link to the electronic supplementary material.

Supplementary Material 1: Full-field and multifocal ERG of Case 1

Supplementary Material 2: Full-field and multifocal ERG of Case 2

Supplementary Material 3: Stacked bar chart showing the number of patients with each type of valve disease, stratified by the presence of EMAP

## Data Availability

Anonymized data used and/or analyzed during the current study are available from the corresponding author on reasonable request.

## Abbreviations

RF: Rheumatic fever
EMAP: Extensive macular atrophy with pseudodrusen–like appearance
SDDs: Subretinal drusenoid deposits
OCT: Optical coherence tomography
FAF: Fundus autofluorescence
BCVA: Best–corrected visual acuity
ETDRS: Early Treatment Diabetic Retinopathy Study
RPE: Retinal pigment epithelium
BM: Bruch’s membrane
cRORA: Complete retinal pigment epithelium and outer retinal atrophy
iRORA: Incomplete retinal pigment epithelium and outer retinal atrophy
NIR: Near–infrared reflectance
ERG: Electroretinogram
BP: Benzathine penicillin
AMD: Age–related macular degeneration
SD: Standard deviation
IQR: Interquartile range
CI: Confidence interval
SW: AF–Short–wavelength autofluorescence
