# Supplementary figures and images for: Assessing the prevalence of extensive macular atrophy with pseudodrusen-like appearance in patients with rheumatic fever-associated valvular heart disease: a cross-sectional study
Source: Int J Retina Vitreous. 2026 Feb 2;12:31. doi: 10.1186/s40942-026-00805-6 (PMC12892698; doi:10.1186/s40942-026-00805-6)

## Case 2

### Dark-adapted

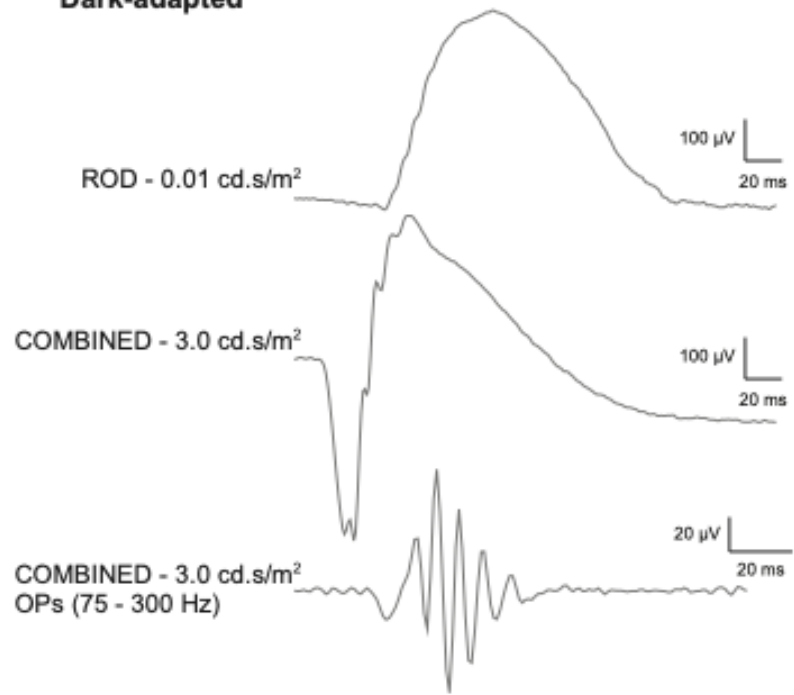

OD

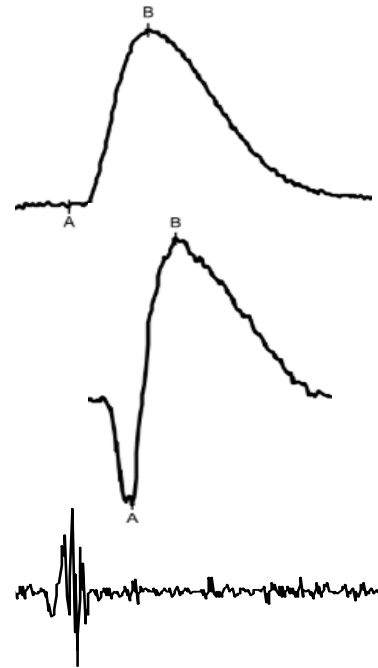

OS

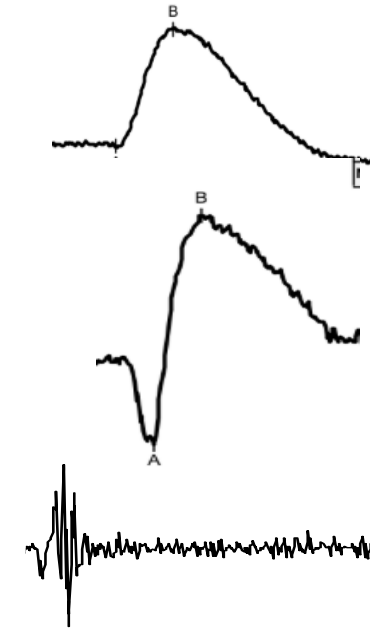

### Light-adapted (30 cd.s/m<sup>2</sup>)

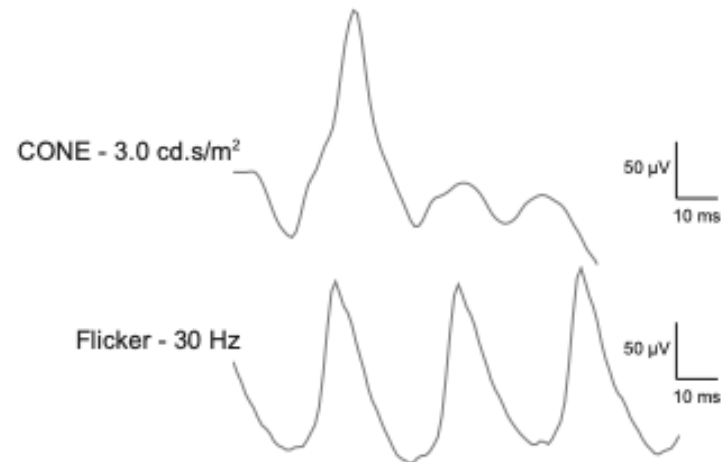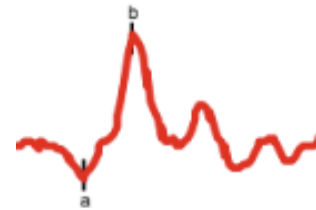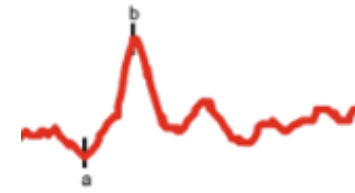

Control

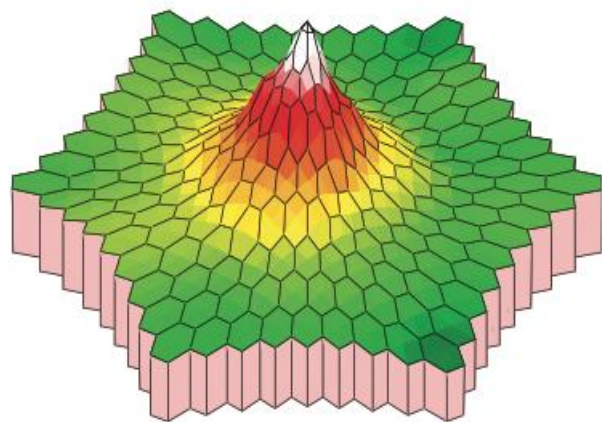

OD

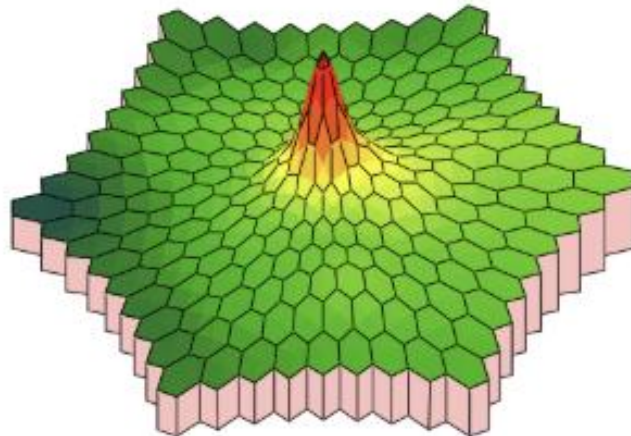

OS

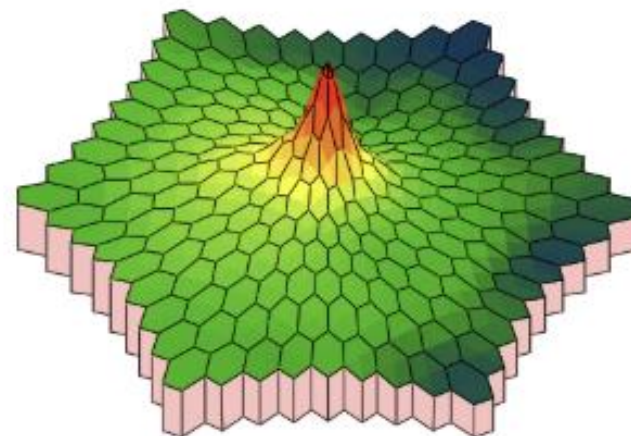

Supplement: Supplementary file 1 — Supplementary Material 1: Full-field and multifocal ERG of Case 1 [file 40942_2026_805_MOESM1_ESM.pdf]

# Case 1

### Dark-adapted

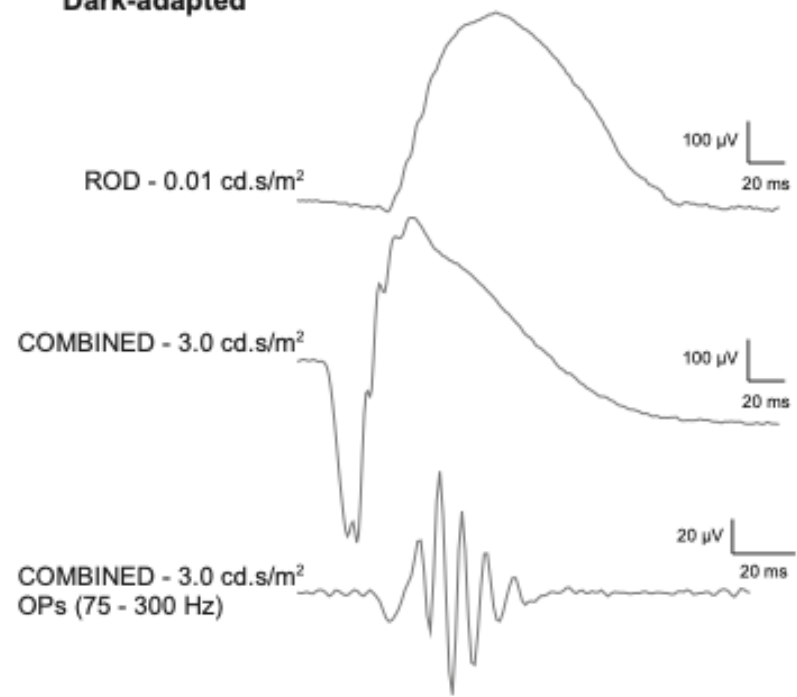

### Light-adapted (30 cd.s/m<sup>2</sup>)

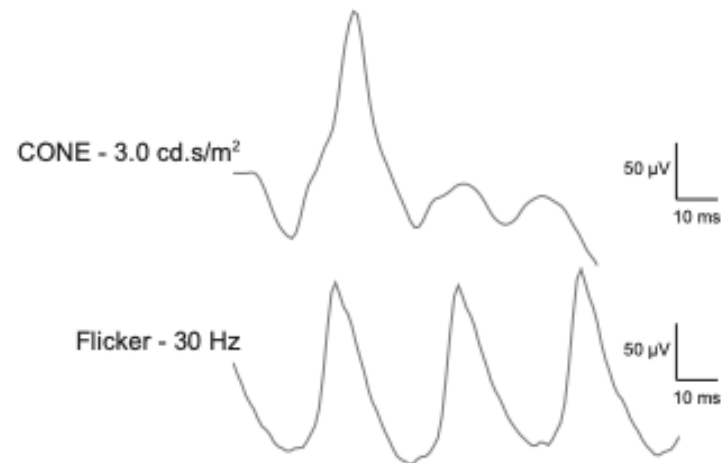

### OD

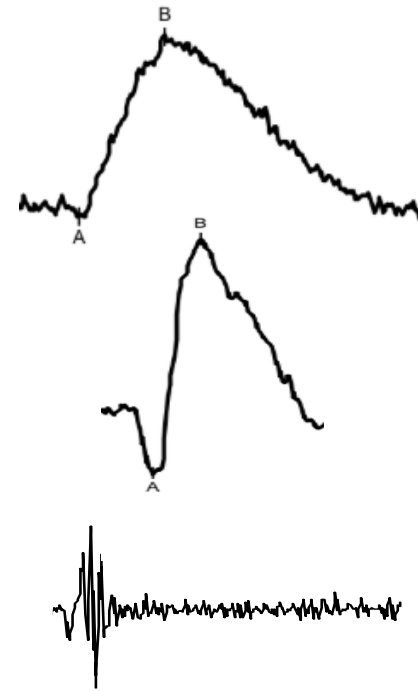

### OS

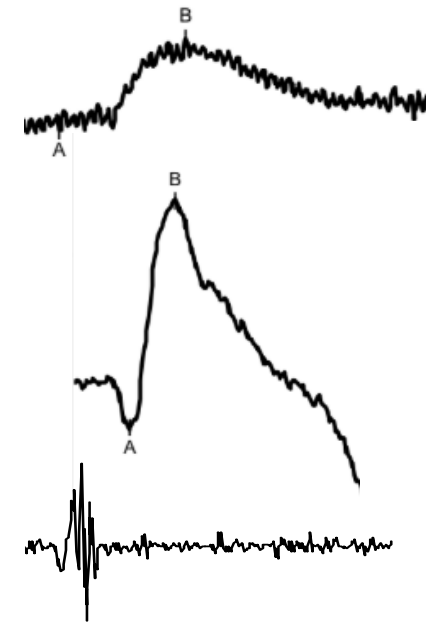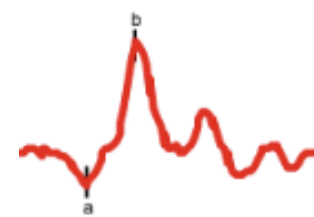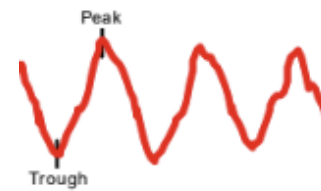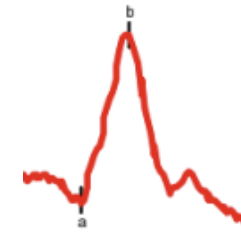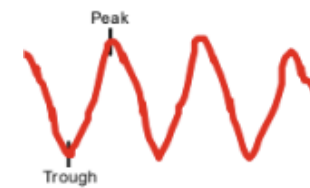

Control

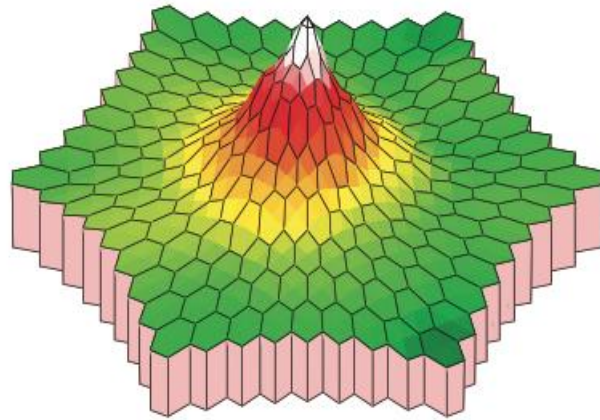

OD

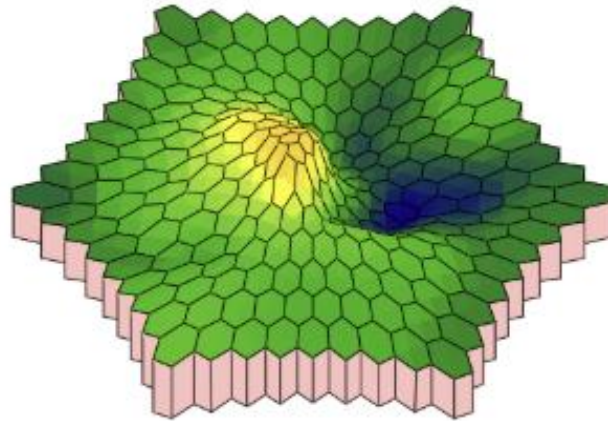

OS

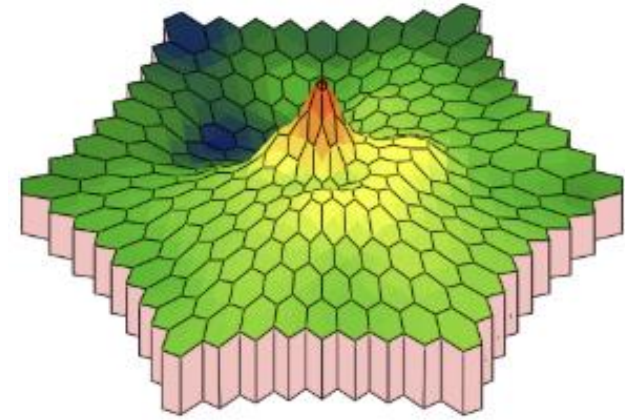

Supplement: Supplementary file 3 — Supplementary Material 3: Stacked bar chart showing the number of patients with each type of valve disease, stratified by the presence of EMAP [file 40942_2026_805_MOESM3_ESM.pdf]
