# Supplementary material for: Assessing the prevalence of extensive macular atrophy with pseudodrusen-like appearance in patients with rheumatic fever-associated valvular heart disease: a cross-sectional study
Source: Int J Retina Vitreous. 2026 Feb 2;12:31. doi: 10.1186/s40942-026-00805-6 (PMC12892698; doi:10.1186/s40942-026-00805-6)

Supplementary File 3 - Stacked bar chart showing the number of patients with each type of valve disease, stratified by the presence of EMAP.

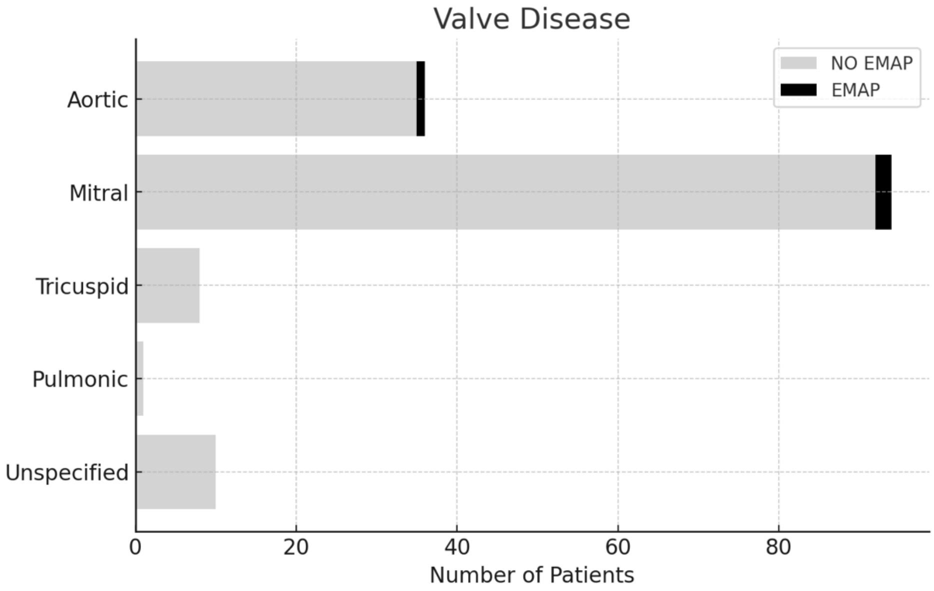

Supplement: Supplementary file 2 — Supplementary Material 2: Full-field and multifocal ERG of Case 2 [file 40942_2026_805_MOESM2_ESM.pdf]
